# The role of pro-inflammatory cytokines and immune cells in colorectal carcinoma progression

**DOI:** 10.3892/ol.2013.1176

**Published:** 2013-02-05

**Authors:** HUANRAN LIU, ZHEN ZHANG, TAKAFUMI TABUCHI, SHOUYU WANG, JIANGNING WANG

**Affiliations:** 1 Department of Surgery, First Affiliated Hospital of Dalian Medical University, Dalian 116011, P.R. China;; 2 Department of Surgery, Kasumigaura Hospital, Tokyo Medical University, Ibaragi 300-0395, Japan;; 3 Department of Reparative and Reconstructive Surgery, Luhe Hospital, Capital Medical University, Beijing 101100, P.R. China

**Keywords:** cytokine, colorectal carcinoma, granulocyte/lymphocyte ratio, interleukin-6, granulocyte colony-stimulating factor, macrophage-colony stimulating factor

## Abstract

Cytokines exhibit a pleiotropic effect in the regulation of the immune cell function, tumor growth and antitumor immune responses. A total of 30 patients with colorectal carcinoma were enrolled on this study and their levels of interleukin (IL)-1β, IL-6, IL-8, tumor necrosis factor (TNF)-α, serum granulocyte colony-stimulating factor (sG-CSF) and serum macrophage colony-stimulating factor (sM-CSF) were measured preoperatively using ELISA. Tumor-infiltrating granulocyte (TIG), tumor-associated macrophage (TAM), G-CSF and M-CSF expression in tumor cells were examined using immunostaining. This study revealed abnormal levels of cytokines in patients, including IL-1β (1/30, 3.3%), IL-6 (16/30 53.3%), IL-81 (15/30, 50%), TNF-α (4/21, 19%), sG-CSF (17/30, 56.7%) and sM-CSF (4/21, 19%). There was a positive linear correlation between IL-6 and sM-CSF (P=0.017, R=0.517). sG-CSF was significantly associated with a deeper tumor invasion (P=0.039) and a more advanced tumor stage (P=0.023). The granulocyte/lymphocyte (G/L) ratio was associated with abnormal levels of sG-CSF. Logistic univariate analysis revealed that TIGs were a risk factor for lymph node metastasis (0.019) and TAMs were a risk factor for depth of invasion (0.029), but this was not confirmed in logistic multivariate analysis. In conclusion, IL-6, IL-8, sM-CSF and sG-CSF may indirectly promote tumor growth, progression and metastasis by changing the leukocyte populations in the blood and the tumor microenvironment.

## Introduction

Cytokines exhibit pleiotropic effects and are crucial for the regulation of cell growth and differentiation. Previous studies have evaluated the cytokine network, which is involved in the local inflammatory and immune responses against tumors (1–4). Cytokines from tumors may either regulate tumor growth or modify the antitumor immune responses (5–8). Tumor and immune cells are capable of producing cytokines, however the key difference between these two cell types is that cytokines produced by tumor cells are not regulated.

Tumor cells are known to produce various cytokines and chemokines that attract leukocytes and promote their transformation into one of the diverse leukocyte populations, mainly granulocytes, monocytes/macrophages, dendritic cells (DCs) and lymphocytes. Subsequently, each population is able to produce an array of cytokines to allow it to escape the immune control of the host (9,10). Cytokines and chemokines from tumor cells also genetically alter epithelial cells and a variety of ‘normal’ cells, including endothelial cells, which form the tumor vasculature, fibroblasts and inflammatory cells (e.g., lymphocytes, macrophages, mast cells and granulocytes), thus building a supportive microenvironment (11,12).

In the present study, we measured the level of pro-inflammatory cytokines, tumor infiltrating granulocytes (TIGs) and tumor-associated macrophages (TAMs), determined the granulocyte/lymphocyte (G/L) ratio and examined the expression of granulocyte colony-stimulating factor (tG-CSF) and macrophage colony-stimulating factor (tM-CSF) in tumor cells to evaluate their role in tumor progression.

## Patients and methods

### 

#### Patient and tumor specimens

A total of 30 patients with primary colorectal carcinoma, who underwent curative surgical treatment between January 2008 and December 2011 at the Department of Surgery, First Affiliated Hospital of Dalian Medical University, Dalian, China, were included in the study. All patients received fasting hemospasia to determine the quantity of cytokines preoperatively. The tumors were histopathologically classified according to the 1997 tumor node metastasis (TNM) classification, as recommended by the International Union Against Cancer (5th edition). None of the patients had received chemotherapy or radiotherapy prior to surgery.

#### ELISA

The levels of immunoreactive serum G-CSF (sG-CSF), sM-CSF, interleukin (IL)-1β, IL-6, IL-8 and tumor necrosis factor (TNF)-α were measured using ELISA. The following commercially available ELISA kits were used: IL-1β, IL-6, TNF-α, M-CSF and G-CSF (R&D systems, Minneapolis, MN, USA) and IL-8 (Biosource Europe S.A., Nivelles, Belgium). The procedures for the cytokine assays were carried out according to the manufacturer's instructions.

### Immunohistochemical analysis of tumor tissues

#### Detection of TIGs and TAMs

Immunostaining procedures for TIGs and TAMs were performed using the EnVision™+/HRP method (Dako, Carpinteria, CA, USA) with heat-induced antigen retrieval. Paraffin sections (4 *μ*m) containing the tumor margin were applied. TIGs and TAMs were detected with mouse anti-granulocyte (clone SPM250, 1:50; Spring Bioscience, Fremonet, Germany) and monoclonal mouse anti-CD68 (clone KP1, 1:200; Dako) antibodies. Negative control sections were stained by omitting the primary antibody.

#### Detection of M-CSF expression (tM-CSF) and G-CSF expression (tG-CSF) in tumor cells

tM-CSF and tG-CSF in colorectal carcinomas were detected by immunohisto-chemical staining with monoclonal anti-G-CSF (clone 4-12-2, 1:100; Immuno-Biological Laboratories, Gunma, Japan) and monoclonal anti-M-CSF (clone EP1179Y, 1:100; Epitomics, Burlingame, CA, USA) antibodies. Negative control sections were stained by omitting the primary antibody. Specimens were considered to be positive for tG-CSF and tM-CSF when ≥20% of the tumor cells exhibited positive immunoreactivity.

#### Statistical analysis

All data, including stage of disease and pathological factors, were obtained from clinical and pathological records. Pearson's correlation coefficient and Spearman's correlation were used to assess the correlation between related variables. Significant differences were determined using the Kruskal-Wallis test. Binary logistic regression analysis was employed to determine the significant predictors for lymph node metastasis and depth of tumor invasion. P<0.05 was considered to indicate a statistically significant difference.

## Results

### 

#### Cytokine production in colorectal patients

A total of 30 patients underwent preoperative blood sampling, however the data for sM-CSF and TNF-α were lost for 9 patients. Abnormal levels (a higher than normal value) of IL-1β, IL-6, IL-8, TNF-α, sG-CSF and sM-CSF were detected in 1/30 (3.3%), 16/30 (53.3%), 15/30 (50%), 4/21(19%), 17/30 (56.7%) and 4/21 (19%) cases, respectively.

#### Detection of TIGs and TAMs and their correlation

**Figure 1:**
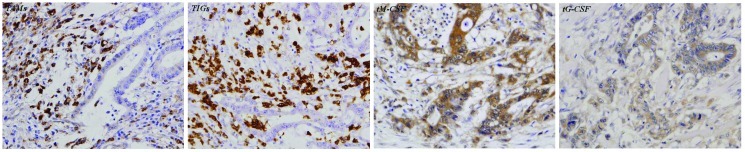
Immunohistochemical assay for TAMs, TIGs, tM-CSF and tG-CSF. TAMs, tumor-associated macrophages; TIGs, tumor-infiltrating granulocytes; tM-CSF, macrophage colony-stimulating factor expression in tumor cells; tG-CSF, granulocyte colony-stimulating factor expression in tumor cells.

The average number of TIGs and TAMs was 47.3±26.1 and 32.5±33.8 per 10×40 hpf, respectively. TIGs were mainly located at the site of tumor invasion, however TAMs were located in areas of tumor invasion and the stroma (Fig. 1). Granulocytes and macrophages in necrotic regions were excluded from the statistical analysis. No significant positive correlation was identified between TIGs and TAMs (P= 0.58, Table I).

#### Correlation between cytokines

We analyzed the correlation between all the cytokines which feature in this study. Pearson's correlation coefficient test revealed that there was a positive linear correlation between IL-6 and sM-CSF (P=0.017, R=0.517) and Spearman's correlation test revealed that there was a significant correlation between IL-6 and sG-CSF (P=0.045, R=0.369). However, no significant correlation was identified between the remaining combinations of cytokines (Table I).

#### Association of G/L ratio with tumor stage and cytokines

**Figure 2:**
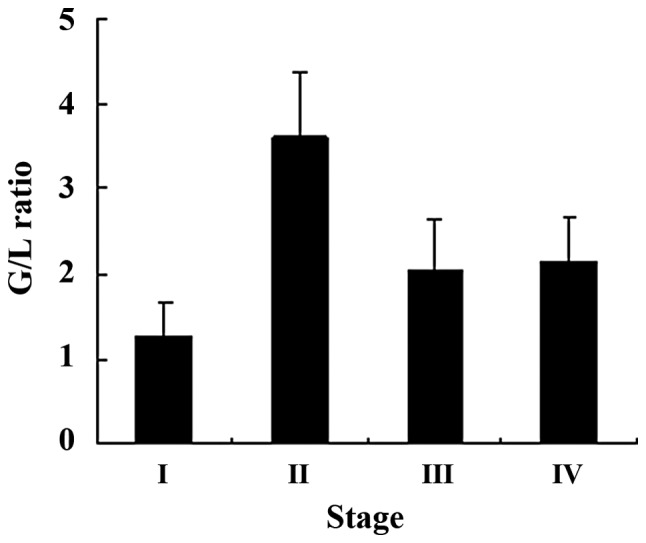
Correlation between the tumor stage and G/L ratio. G/L, granulocyte/lymphocyte.

We investigated the association between the tumor stage/cytokines and the G/L ratio; a significant correlation was identified for tumor stage (P= 0.037, Fig. 2), however, the G/L ratio was not associated with the levels of cytokines (Table I).

#### Association of cytokines with TIGs and TAMs

**Figure 3:**
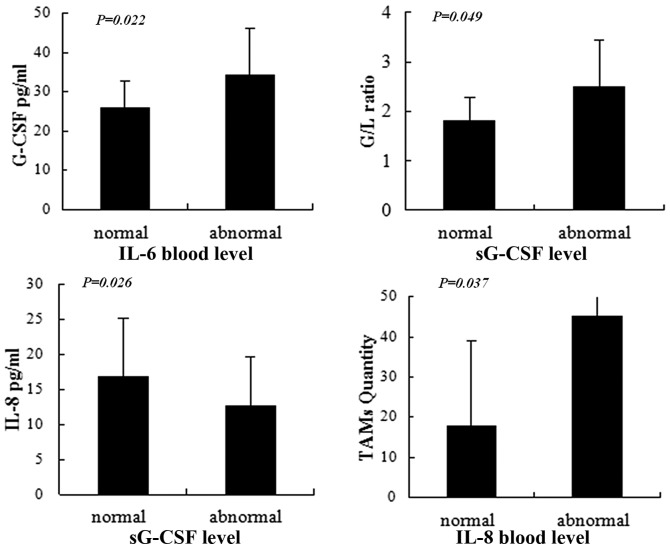
Effects of cytokine levels. Kruskal-Wallis testing revealed that sG-CSF was significantly increased in the abnormal IL-6 level group (P= 0.022); the G/L ratio was associated with abnormal levels of sG-CSF (P=0.049); IL-8 was significantly decreased in the high sG-CSF level group (P= 0.026); TAMs were associated with abnormal levels of IL-8 (P= 0.037). sG-CSF, serum granulocyte colony-stimulating factor; G/L, granulocyte/lymphocyte; IL, interleukin, TAMs, tumor-associated macrophages.

The patients were divided into groups for normal or abnormal cytokine levels. Kruskal-Wallis testing revealed that the level of sG-CSF was significantly increased in the abnormal IL-6 level group (P=0.022), the G/L ratio was significantly increased in the abnormal sG-CSF level group (P=0.049), the level of IL-8 was significantly decreased in the abnormal sG-CSF level group (P=0.026) and TAM levels were significantly increased in the abnormal IL-8 level group (P=0.037; Fig. 3).

#### Association of cytokines, TIGs and TAMs with clinico-pathological factors

Analysis revealed that sG-CSF was significantly associated with the depth of tumor invasion (P=0.039) and a more advanced tumor stage (P=0.023). However, significant correlation was not identified between cytokines and lymph node metastasis and between TIGs or TAMs and tumor stage, depth of invasion and lymph node metastasis (Table II).

#### Detection of tM-CSF and tG-CSF by immunostaining

**Figure 4:**
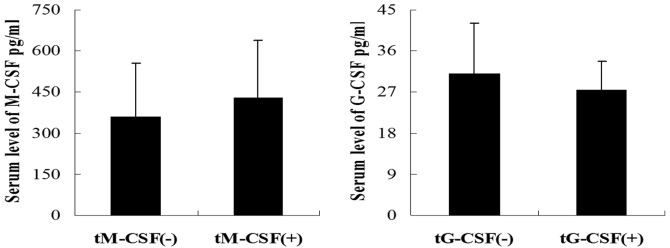
Correlation between CSF expression in tumor cells and the level of serum CSF. There was no significant correlation between tM-CSF and the level of sM-CSF (P=0.442) and between tG-CSF and the level of sG-CSF (P= 0.498). CSF, colony-stimulating factor; tM-CSF, macrophage CSF expression in tumor cells; sM-CSF, serum macrophage CSF; tG-CSF, granulocyte CSF expression in tumor cells; sG-CSF, serum granulocyte CSF.

Immunostaining, which used antibodies against tM-CSF and tG-CSF (Fig. 1), revealed that tM-CSF and tG-CSF in tumor cells was detected in 17/30 (56.7%) and 5/30 (16.7%) of cases, respectively. No significant correlation was identified between CSF expression in tumor cells and the serum levels of CSF for M-CSF (P=0.442) or G-CSF (P=0.498; Fig. 4).

#### Logistic regression analysis of risk factors for lymph node metastasis and depth of tumor invasion

Logistic univariate analysis revealed that TIGs were correlated with lymph node metastasis, however, in logistic multivariate analysis, they were not identified as a significant independent risk factor for lymph node metastasis (P=0.069). Logistic univariate analysis also revealed that levels of TAMs were correlated with depth of invasion, however, in logistic multivariate analysis, they were not identified as an independent risk factor for depth of invasion (P=0.063; Table III).

## Discussion

This study aimed to clarify the correlation between pro-inflammatory cytokines, immune cells and tumors. This has previously been investigated by numerous studies, which showed that cytokines regulate cell growth and, more importantly, cell proliferation, and they may be produced by tumor or immune cells. However, the role of cytokines in inducing granulocytes or macrophages to become involved in the anti-tumor immune response was unclear.

G-CSF is produced by normal monocytes, macrophages and granulocytes. In the present study, we identified abnormal levels of sG-CSF in 17/30 (56.7%) cases, however, only a few cases of G-CSF-producing colorectal carcinomas have ever been reported. We also examined tG-CSF expression in tumor cells using immunohistochemical staining. A reaction for tG-CSF was observed in only 5 cases and this was not associated with the level of sG-CSF (P=0.498). The levels of immunoreactive sM-CSF, IL-1β, IL-6, IL-8 and TNF-α were measured by ELISA and a positive linear correlation was identified between the levels of IL-6 and sM-CSF. The level of sG-CSF was significantly increased in the abnormal IL-6 level group. M-CSF expression in tumor cells was examined using immunostaining and immunoreactivity was observed in 17/30 (56.7%) cases, which is similar to results from previous studies on M-CSF production by tumor cells (13–16). However, only 4 patients (19%) had abnormal levels of sM-CSF and there was no significant correlation between tM-CSF and sM-CSF (P=0.442). A study by Ashizawa *et al*(17) showed that immunohistochemical staining revealed positive results for IL-6 expression in the cytoplasm of colorectal cancer cells in patients with a high serum level of IL-6, however there was no evidence of positive results for IL-6 expression in patients with a normal serum level of IL-6. This suggests that IL-6 may be able to induce the production of CSFs from numerous types of cells, in addition to tumors (16). IL-6 is a pleiotropic cytokine which is secreted by a wide variety of cell types, including lymphocytes, monocytes and tumor cells (17,18). Sato *et al*(19) demonstrated that IL-6, sG-CSF and sM-CSF appear to contribute to neutrophilia in cases of anaplastic thyroid carcinoma. A high count of peripheral neutrophils was correlated with poor prognosis in patients with a variety of cancer types, including breast, head and neck cancer and sarcoma (20–24).

Therefore, we measured the G/L ratio, which represents the relative number of these two major leukocyte populations, indicates fluctuations in their numbers and reveals their potential impact on the progression and prognosis of cancer. Hence, the G/L ratio, which is easily measured in a clinical setting, is a valuable indicator of tumor progression (25) and is useful for selecting patients who are appropriate for surgery (26). Our results revealed that the G/L ratio was significantly associated with a more advanced tumor stage (P=0.037) and it was significantly increased in the abnormal sG-CSF level group. Thus it is hypothesized that sG-CSF, sM-CSF and IL-6 may indirectly assist tumor growth and progression by neutrophilia. Next, we analyzed the correlation between cytokines and tumor features. The results revealed that sG-CSF levels were positively associated with deeper tumor invasion and a more advanced tumor stage.

In carcinogenesis, cytokines and chemokines from tumor cells are able to build a supportive microenvironment and induce inflammatory cells, which contribute to tumor growth, progression and metastasis (27–29). In this study, measuring the quantity of TIGs and TAMs revealed that levels of TIGs were increased with tumor progression, however TAM levels were decreased. Next, the correlation between cytokines and TIGs or TAMs was tested; a significantly positive correlation was identified between IL-8 and TAMs and the IL-8 level was significantly decreased in the high sG-CSF level group. Based on the above-mentioned results for sG-CSF, we hypothesized that IL-8 should decrease with tumor progression, however, the present study failed to demonstrate this. This may be due to the complicated role of TAMs in tumor progression; since TAMs have a dual role in neoplasms, it is difficult to accurately evaluate the correlation between IL-8 and TAMs. IL-8 is known to recruit inflammatory neutrophils and promote the interaction between tumor cells and inflammatory cells (30) and it is also a potent angiogenic and growth factor in malignant tumors (31,32). Therefore, we hypothesized that IL-8 may selectively induce the infiltration of immune cells into tumors, in order to assist tumor progression. The present study evaluated the risk factors for lymph node metastasis and depth of invasion by logistic regression analysis. Univariate analysis revealed that TIGs were correlated with lymph node metastasis, however, logistic multivariate analysis determined that P=0.069, which is close to 0.05. Further univariate analysis revealed that TAMs were correlated with depth of invasion and multivariate analysis revealed that P=0.063, which is also close to 0.05. It is possible that significant results were not obtained due to the small sample size, however, we believe that TIGs and TAMs should be risk factors for lymph node metastasis and depth of invasion, respectively.

Due to the pleiotropy of cytokines, it is difficult to confirm the roles of cytokines in the growth and progression of tumors. However, the present study reveals a correlation between tumors and immune cells, which secrete cytokines, and this suggests that cytokines may indirectly promote the growth, progression and metastasis of tumors..

## Figures and Tables

**Table I:** Correlation between cytokines and G/L ratio.

Variable	IL-6	sM-CSF	sG-CSF	IL-8	IL-1β	TNF-α	G/L ratio	TIGs
sM-CSF								
R	0.517^b^							
P-value	0.017							
sG-CSF								
R	0.369^a^							
P-value	0.045	0.56						
IL-8								
R								
P-value	0.63	0.75	0.37					
IL-1β								
R								
P-value	0.90	0.99	0.56	0.43				
TNF-α								
R								
P-value	0.44	0.69	0.44	0.89	0.62			
G/L ratio								
R								
P-value	0.24	0.89	0.45	0.58	0.65	0.67		
TIGs								
R						0.43^a^		
P-value	0.945	0.320	0.364	0.445	0.297	0.050	0.888	
TAMs								
R				0.32^a^				
P-value	0.64	0.75	0.33	0.09	0.67	0.49	0.40	0.579

a Spearman's test;

b Pearson's test. There was a significant correlation between IL-6 and sM-CSF (P=0.017) and between TAMs and TIGs (P=0.024). IL, interleukin; sM-CSF, serum macrophage colony-stimulating factor; sG-CSF, serum granulocyte colony-stimulating factor; TNF-α, tumor necrosis factor-α; G/L ratio, granulocyte/lymphocyte ratio; TIGs, tumor-infiltrating granulocytes; TAMs, tumor-associated macrophages.

**Table II:** Association of cytokines with clinicopathological factors.

Variable	Cytokines according to tumor features (pg/ml)								

	Lymph node metastasis			Depth of invasion			Tumor stage		
		
	Absent	Present	P-value	Under muscle layer^a^	Beyond muscle layer	P-value	Stage 0 and I	Stage II, III and IV	P-value
sG-CSF	26.5±7.6	32.2±11.2	NS	23.9±4.1	31.4±11.1	0.039	22.7±4.1	32.4±10.7	0.023
sM-CSF	504±241.4	359.2±172.5	NS	407.3±208.82	399±138.9	NS	470.7±295.1	389.0±188.9	NS
IL-1β	0.32±0.2	0.28±0.2	NS	0.4±0.3	0.2±0.1	0.086	0.35±0.2	0.28±0.2	NS
IL-6	23.3±62.5	5.0±7.1	NS	2.4±1.4	12.9±17.2	NS	2.52±2.1	12.48±38.4	NS
IL-8	10.9±4.2	16.1±14.4	NS	9.9±2.0	16.0±8.9	NS	10.2±2.5	15.6±13.6	NS
TNF-α	1.0±0.6	1.3±0.4	NS	1.0±0.3	1.2±0.6	NS	1.0±0.5	1.2±0.8	NS
TIGs	33.9±12.5	53.1±22.9	0.065	40.3±16.5	49.5±23.1	NS	40.6±18.2	50.2±21.9	NS
TAMs	32.4±16.8	32.5±28.3	NS	42.6±22.5	29.4±25.5	NS	46.2±39.0	26.6±20.4	NS

Results are presented as mean ± SD.

a Includes muscle layer. sG-CSF was significantly associated with deeper tumor invasion (P=0.039) and more advanced tumor stage (P=0.023, Rs=0.375).sG-CSF, serum granulocyte colony-stimulating factor; sM-CSF, serum macrophage colony-stimulating factor; IL, interleukin; TNF-α, tumor necrosis factor-α; TIGs, tumor-infiltrating granulocytes; TAMs, tumor-associated macrophages; NS, not significant.

**Table III:** Logistic regression analysis of risk factors for lymph node metastasis and depth of invasion.

		Multivariate	
Variable	Univariate P-value	P-value	RR (95% CI)
Lymph node metastasis^a^			
G-CSF	0.269		
M-CSF	0.124		
IL-1β	0.731		
IL-6	0.15		
IL-8	0.703		
TNF-α	0.452		
TIGs	0.019	0.069	1.087 (0.994–1.188)
TAMs	0.353		
sG-CSF	0.517		
sM-CSF	0.676		
Depth of invasion^b^			
G-CSF	0.083	0.071	1.301 (0.978–1.733)
M-CSF	0.939		
IL-1β	0.083		
IL-6	0.517		
IL-8	0.567		
TNF-α	0.59		
TIGs	0.262		
TAMs	0.029	0.063	0.64 (0.927–1.002)
sG-CSF	0.619		
sM-CSF	0.422		

a -2 Log likelihood = 17.811, Nagel kerke R^2^ = 0.422;

b -2 Log likelihood = 9.362, Nagel kerke R^2^ = 0.659. CI, confidence interval; G-CSF, granulocyte colony-stimulating factor; M-CSF, macrophage colony-stimulating factor; IL, interleukin; TNF-α, tumor necrosis factor-α; TIGs, tumor-infiltrating granulocytes; TAMs, tumor-associated macrophages; sG-CSF, serum G-CSF; sM-CSF, serum M-CSF.
